# Maternal salt and fat intake causes hypertension and sustained endothelial dysfunction in fetal, weanling and adult male resistance vessels

**DOI:** 10.1038/srep09753

**Published:** 2015-05-08

**Authors:** Clint Gray, Claudia J. Harrison, Stephanie A. Segovia, Clare M. Reynolds, Mark H. Vickers

**Affiliations:** 1 Liggins Institute and Gravida: National Centre for Growth and Development, University of Auckland, New Zealand

## Abstract

Maternal salt and fat intake can independently programme adult cardiovascular status, increasing risk of cardiovascular disease in offspring. Despite its relevance to modern western-style dietary habits, the interaction between increased maternal salt and fat intake has not been examined. Female virgin Sprague-Dawley rats were fed, a standard control diet (CD) (10% kcal fat, 1% NaCl), High-fat diet (HF) (45% kcal fat, 1% NaCl), High-salt diet (SD) (10% kcal fat, 4% NaCl), High-fat high-salt diet (HFSD) (45% kcal fat, 4% NaCl) prior to pregnancy, during pregnancy and throughout lactation. Fetal, weanling and adult vessels were mounted on a pressure myograph at fetal day 18, weaning day 21 and day 135 of adulthood. Increased blood pressure in SD, HFD and HFSD male offspring at day 80 and 135 of age was consistent with perturbed vascular function in fetal, weanling and adult vessels. Maternal salt intake reduced EDHF and calcium-mediated vasodilation, maternal fat reduced NO pathways and maternal fat and salt intake, a combination of the two pathways. Adult offspring cardiovascular disease risk may, in part, relate to vascular adaptations caused by maternal salt and/or fat intake during pregnancy, leading to persistent vascular dysfunction and sustained higher resting blood pressure throughout life.

Hypertension affects more than 25% of adults aged ≥30 years globally^1^. The global incidence of non-communicable disease (NCD), specifically, obesity and cardiovascular disease (CVD) is increasing year on year and these disease states have largely been attributed to lifestyle factors such as diet and a sedentary existence^2^. Moreover, Western diets are commonly associated with high fat, sugar and salt intake, all of which have been associated with the current exponential increase in NCD observed over the past four decades^2^,^3^,^4^. The prevalence of obesity, especially in women prior to and/or during pregnancy, is a major health concern and there is now a large body of evidence showing that maternal obesity can program later life disease in adult offspring^5^. Despite the current prevalence of excessive fat and salt intake in the modern Western diet, the developmental programming effects of these maternal dietary components, fed in combination, have not yet been examined.

It is widely accepted that maternal dietary intake and nutritional environment during fetal development and early life have long-term implications for offspring cardiovascular status and predisposition to CVD during adulthood^6^,^7^,^8^. Developmental programming of CVD risk has been observed in many studies utilising animal models of maternal micro- and macronutrient restriction and excess. Our group, using models of overnutrition have reported that excessive maternal fat, fructose and salt intake can lead to adult obesity, insulin resistance, endothelial dysfunction, hypertension and predisposition to CVD in offspring^9^,^10^,^11^,^12^. Hypertension and subsequent increased risk of CVD caused by maternal overnutrition in the form of excessive fat consumption have been shown to perturb vascular function by reducing nitric oxide synthase activity and reduced vascular vasoresponsiveness in offspring. Similarly, excessive maternal salt intake has also been shown to cause endothelial dysfunction, reduced angiogenesis, perturbed branching organogenesis and hypertension in adult offspring^13^,^14^,^15^. The effects of programming on offspring’s vascular development and programmed cardiovascular phenotype during adulthood are thought to be permanent artefacts of *in utero* development stemming from suboptimal maternal nutrition during fetal development leading to a persistent higher blood pressure throughout life and an increased risk of CVD during adulthood^14^,^15^,^16^.

In the current study we aimed to investigate whether maternal fat and salt in combination play a role in the development of hypertension and perturbed vascular function during offspring later life and whether these alterations were present during development and early life. To examine the effect of increased salt intake on acute vascular function in normotensive rats or the effects of maternal salt and fat on the developing offspring, investigators have typically used experimental diets containing between 4–8% NaCl and/or 45% kcals derived from fat. Studies have shown an increase in offspring blood pressure in response to maternal salt-feeding at 4 and 8% salt diets but not always at 4%^13^,^14^,^15^,^16^,^17^. We hypothesised that feeding a maternal diet high in fat (45%) and/or salt (4%) to female rats 21 days prior to pregnancy, during and throughout lactation would lead to perturbed vascular function in the fetus and young offspring, leading to a hypertensive phenotype in adult offspring. Furthermore, the combination of fat and salt during pregnancy would exacerbate subsequent male offspring vascular function and blood pressure.

## Methods

### Animal ethics

All procedures described were approved and performed in accordance with relevant guidelines and regulations outlined by the Animal Ethics Committee at the University of Auckland (Approval R1069).

### Experimental protocol

80 female virgin Sprague Dawley rats were fed *ad-libitum* from weaning until day 90 and maintained at 25 °C and a 12 h light: 12 h darkness cycle. Female rats were then randomly assigned to four dietary groups and fed experimental diets *ad-libitum* for 21 days prior to pregnancy. Experimental groups were fed either (1) a control (CON, n = 20) purified standard diet (1% NaCl, 10% kcal from fat Research Diets NJ USA); (2) a purified 4% NaCl diet, 10% kcal from fat (SD, n = 20); (3) a purified 45% kcal fat diet +1% NaCl (HF, n = 20) and (4) a purified 45% kcal from fat +4% NaCl diet (HFS, n = 20) *ad-libitum* throughout pregnancy and lactation. Female rats (115 days of age +/- 2, (n = 60)) were time-mated using an estrus cycle monitor (Fine Science Tools, USA). Day 1 of pregnancy was determined by the presence of spermatozoa after a vaginal lavage. Females were then individually housed and continued on the experimental diets throughout gestation and lactation. At day 18 of gestation a sub-cohort of pregnant dams (n = 6/group) were culled and fetuses were collected for vessel dissection, pressure myography and physiological analysis. Remaining dams were allowed to give birth. All pups were weighed, sexed and had body lengths recorded. Litter size was randomly adjusted to 8 pups (4 male, 4 female) to ensure standardized nutrition until weaning. Pups not allocated to litters were killed by decapitation. At weaning, male offspring were housed two/cage (two per litter/treatment/maternal background) and fed a standard chow diet *ad-libitum* until day 140 (Diet 2018, Harlan, Oxon, UK). A minimum of eight offspring/group/sex were investigated. At postnatal day 80 and 135, systolic blood pressure was measured *via* tail cuff plethysmography (Model 179, IITC Life Science Inc, USA). Due to logistical constraints, only male offspring were used in the present study. All offspring were collected from different dams/litters, n = 8 (fetal & weanling) or n = 10 (adult offspring) represents eight or ten offspring from eight or ten different litters. At postnatal day 140, animals were fasted overnight and following anaesthesia with sodium pentobarbitone (60 mg/kg, IP) killed by decapitation.

We utilised a 4% purified salt diet so as not to induce spontaneous hypertension during pregnancy. Purified diets were used to ensure balanced isocaloric intakes without inconsistencies in micro- and macronutrient levels.

### Blood pressure measurements

Systolic blood pressure (SBP) at day 80 and 135 was recorded by tail cuff plethysmography (Model 179 (NW20), IITC, Life Science, Woodland Hills, CA) to assess any age-related changes in blood pressure as previously described^18^. Briefly, rats were placed in a clear plastic tube in a pre-warmed room (25–28 °C). After acclimatisation (10–15 min) the inflation cuff was placed on the base of the tail and inflated to 240 mmHg. Pulse was recorded during deflation at a rate of 3 mmHg/s and appearance of a defined pulse was used to determine SBP. Three SBP recordings/animal were taken and averaged. The coefficient of variation for repeated measurements was <5%.

### Vascular studies

Second order femoral arteries (<200 μm) were analysed in fetuses and 3^rd^ order mesenteric vessels (<250 μm) were analysed in weanlings and adult offspring. All connective tissue was removed using a dissecting microscope and placed in a dissecting dish containing physiological salt solution (PSS) (119 mM NaCl, 4.7 KCl, 2.5 CaCl_2_, 24 NaHCO_3_, 1.18 KH_2_PO_4_, 1.2 MgSO_4_, 0.01 EDTA, 5.5 glucose) and placed on ice until mounting. Vessels were analysed using a pressure myograph system (Living System, Burlington, VT, USA). Vessels were placed on two glass micro-cannulae and secured with nylon suture. Intraluminal pressure was then increased to 100 mmHg and the vessels adjusted to ensure it was parallel and not stretched. To ensure vessels were viable, five one minute washes with PSS and pre-constriction with phenylephrine (PE) (concentration equal to 80% of maximal response; pEC80) were performed. Vessels were pre-constricted to pEC80 and vessels failing to consistently reproduce constriction (pEC80) were also discarded and substituted with freshly excised tissue.

All subsequent vascular studies were studied in vessel segments pressurized to 80 mmHg following the equilibration period of 20 minutes or cessation of basal vascular activity, at 37 °C in PSS gassed with a mixture of 95% O_2_ and 5% CO_2_. Cumulative concentration response curves were constructed for the α1-adrenoceptor agonist PE (1 nM to 100 μM). Changes in diameter at each PE concentration were compared to initial vessel diameter as % constriction, and then normalised as % maximum constriction. Following pre-constriction with PE ^–^log concentration equal to 80% of maximal response, cumulative concentration curves were constructed with the endothelium-dependant vasodilator acetylcholine (ACh; 0.1 nM to 1 mM). Changes in diameter at each ACh concentration were compared to initial vessel diameter after pre-constriction with PE, and normalised as % relaxation. The present study investigated mediators of endothelium-dependent relaxation and endothelial-independent pathways. To block soluble guanylyl cyclase activity (NO production), non-specific NO synthase inhibitor L-NG-Nitroarginine Methyl Ester (L-NAME, 100 μM) and soluble guanylyl cyclase inhibitor 1H-[1,2,4]oxadiazolo[4,3-a]quinoxalin-1-one (ODQ, 5 μM) were used. Indomethacin (INDO, 10 μM) was used to block cyclooxygenase pathway. The role of gap junctions and EDHF activity was investigated using the gap junction inhibitor GAP-27 (GAP, 100 μM), intermediate-conductance Ca2^+-^activated K^+^ channel blocker TRAM-34 (1 μM) and ATP-type Ca^2+-^activated K^+^ channel blocker apamin (3 μM).

### Determination of vascular reactive oxygen species

Total relative oxygen species in mesenteric vessel segments were measured with Oxiselect in vitro ROS/RNS assay kits (Cell Biolabs, Inc, San Diego, CA) following the manufacturer’s instructions.

### RNA isolation

RNA was isolated from mesenteric vessel tissue using RNeasy^®^ mini kit (QIAGEN, Hilden, Germany) and cDNA synthesized from 2 μg of RNA using SuperScript^®^ VILO^™^ cDNA Synthesis Kit (Invitrogen^™^; Life Technologies Corporation, California, USA).

### Gene expression analysis

PCR analysis for offspring cardiac derived cyclooxygenase 1 & 2 (COX-1, COX-2), endothelial nitric oxide synthase (eNOS) expression was carried out by using Pre-Developed TaqMan^®^ Assay Reagent Kits in the ABI 7900HT Fast Real-Time PCR System (Applied Biosystems). Relative amounts of genes were normalized to the geometric mean of reference genes, cyclophilin A (cycA) and hypoxanthine phosphoribosyltransferase (HPRT). To control for between-sample variability, mRNA levels were normalized to the geometric mean of for each sample by subtracting the Ct of controls from the Ct for the gene of interest producing a Ct value. The ΔCt for each treatment sample was compared to the mean ΔCt for control samples using the relative quantification 2-(ΔΔCt) method to determine fold-change.

### Statistical analysis

Two-way repeated measures factorial ANOVA with Bonferroni *post-hoc* test where appropriate and concentration-relaxation curves were constructed using Prism software (GraphPad Software Inc., La Jolla, CA, USA.). Two-way analysis of variance (ANOVA) was used to determine significant differences and maternal diet and their interactions were considered as independent variables. When significance (p < 0.05) was indicated, *post-hoc* Holm-Sidak test analysis was used to determine which values were significantly different from each other. A probability of p < 0.05 was accepted as statistically significant.

## Results

### Systolic blood pressure (SBP) recordings

**Figure 1:**
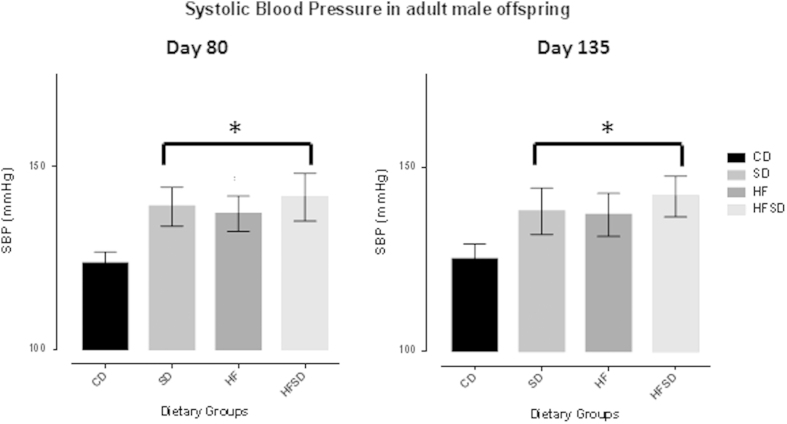
(**a**) Systolic blood pressure (SBP) at postnatal day 80 in male offspring as quantified *via* tail-cuff plethysmography. *p < 0.001 for SD, HF & HFSD versus CON. All data are means ± SEM, n = 10/group. Different dietary groups are defined as; Control group = CD, Salt group = SD, High fat group = HF and Salt and Fat combined group = HFSD, n = 10/group.(**b**). Systolic blood pressure (SBP) at postnatal day 135 in male offspring as quantified *via* tail-cuff plethysmography. *p < 0.001 for SD, HF & HFSD versus CON. All data are means ± SEM, n = 10/group. Different dietary groups are defined as; Control group = CD, Salt group = SD, High fat group = HF and Salt and Fat combined group = HFSD, n = 10/group.

An effect of salt and fat and fat/salt combined was observed (P < 0.001^in both cases^) on SBP in adult offspring at day 80 and day 135. SBP at day 80 was significantly increased in SD, HF and HFSD offspring when compared to CD. On average, at day 80 and day 135, SBP in HFSD offspring were >5% higher than that of SD and HF offspring SBP, However, these differences were not significant. All offspring groups expressed the same degree of basal systolic blood pressure increase without an effect of age at this stage (day 80 vs. day 135 SBP) (Fig. 1a,b).

## Endothelium-dependent dilation

### Vessel responsiveness to PE in fetal, weanling and adult offspring

**Figure 2:**
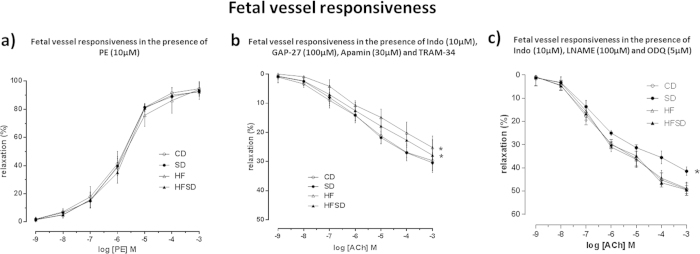
(**a**) Fetal offspring mesenteric vessel responsiveness following phenylephrine (PE) treatment, measured as % change from initial resting diameter and normalised as % maximum constriction in CD , SD , HF and HFSD fetuses. All data are means ± SEM. Different dietary groups are defined as; Control group = CD, Salt group = SD, High fat group = HF and Salt and Fat combined group = HFSD, n = 10/group. (**b**) Fetal femoral vessel responsiveness following cumulative addition of vasodilator ACh measured as % change from initial resting diameter after pre-constriction with PE (10 μM) in the presence of TRAM-34 (1 μM), Apamin (3 μM) and INDO (10 μM). All data are means ± SEM. * p < 0.001 for HF versus CD. ** p < 0.001 for HFSD versus CD. Different dietary groups are defined as; Control group = CD, Salt group = SD, High fat group = HF and Salt and Fat combined group = HFSD, n = 8/group. (**c**) Fetal femoral vessel responsiveness following cumulative additions of vasodilator ACh measured as % change from initial resting diameter after pre-constriction with PE (10 μM) in the presence of L-NAME (100 μM), INDO (10 μM) and ODQ (5 μM). All data are means ± SEM. * p < 0.001 for SD versus all other groups. Different dietary groups are defined as; Control group = CD, Salt group = SD, High fat group = HF and Salt and Fat combined group = HFSD, n = 8/group.

**Figure 3:**
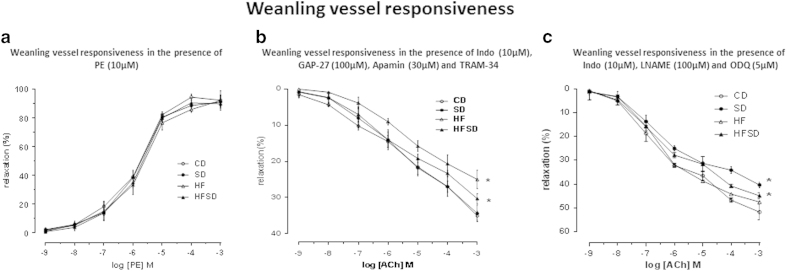
(**a**) Weanling offspring mesenteric vessel responsiveness following phenylephrine (PE) treatment, measured as % change from initial resting diameter and normalised as % maximum constriction in CD, SD, HF and HFSD weanling male offspring. All data are means ± SEM. Different dietary groups are defined as; Control group = CD, Salt group = SD, High fat group = HF and Salt and Fat combined group = HFSD, n = 10/group. (**b**) Weanling mesenteric vessel responsiveness following cumulative addition of vasodilator ACh measured as % change from initial resting diameter after pre-constriction with PE (10 μM) in the presence of TRAM-34 (1 μM), Apamin (3 μM) and INDO (10 μM). All data are means ± SEM. * p < 0.001 for HF versus CD. ** p = 0.04 for HFSD versus CD. Different dietary groups are defined as; Control group = CD, Salt group = SD, High fat group = HF and Salt and Fat combined group = HFSD, n = 8/group. (**c**) Weanling mesenteric vessel responsiveness following cumulative additions of vasodilator ACh measured as % change from initial resting diameter after pre- constriction with PE (10 μM) in the presence of L-NAME (100 μM), INDO (10 μM) and ODQ (5 μM). All data are means ± SEM. * p < 0.001 for SD versus all other groups. Different dietary groups are defined as; Control group = CD, Salt group = SD, High fat group = HF and Salt and Fat combined group = HFSD, n = 8/group.

**Figure 4:**
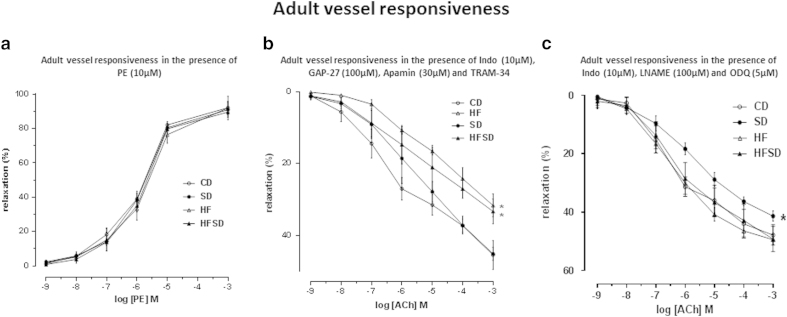
(**a**) Adult offspring mesenteric vessel responsiveness following phenylephrine (PE) treatment, measured as % change from initial resting diameter and normalised as % maximum constriction in CD, SD, HF and HFSD adult male offspring. All data are means ± SEM. Different dietary groups are defined as; Control group = CD, Salt group = SD, High fat group = HF and Salt and Fat combined group = HFSD, n = 10/group. (**b**) Adult offspring mesenteric vessel responsiveness following cumulative addition of vasodilator ACh measured as % change from initial resting diameter after pre-constriction with PE (10 μM) in the presence of TRAM-34 (1 μM), Apamin (3 μM) and INDO (10 μM). All data are means ± SEM. * p < 0.001 for HF versus CD. ** p = 0.001 for HFSD versus CD. Different dietary groups are defined as; Control group = CD, Salt group = SD, High fat group = HF and Salt and Fat combined group = HFSD, n = 10/group. (**c**) Adult mesenteric vessel responsiveness following cumulative additions of vasodilator ACh measured as % change from initial resting diameter after pre-constriction with PE (10 μM) in the presence of L-NAME (100 μM), INDO (10 μM) and ODQ (5 μM). All data are means ± SEM. * p < 0.001 for SD versus all other groups. Different dietary groups are defined as; Control group = CD, Salt group = SD, High fat group = HF and Salt and Fat combined group = HFSD, n = 10/group.

PE produced a concentration-dependant vasoconstriction in all vessels. Mesenteric vessel responsiveness to PE was not significantly impaired across groups at any time point (fetal, weanling and adult) (Figs. 2a,3a,4a)

### ACh-induced relaxation in vessels incubated with L-NAME, TRAM-34, Apamin, GAP-27

To assess the contribution of prostanoid pathways of vasodilation, fetal, weanling and adult vessels were incubated with LNAME (100 μM), TRAM-34 (1 μM), Apamin (3 μM), GAP-27 (100 uM). Following pre-constriction with PE, no differences were observed between groups following ACh-induced vasodilation (Supplementary Figure 1b,2b,3b).

### ACh-induced relaxation in vessels incubated with TRAM-34, Apamin, GAP-27 & INDO

#### Fetal vessels

Fetal vessels in the presence of TRAM-34 (1 μM), Apamin (3 μM), GAP-27 (100 uM) and INDO (10 μM) induced a significant reduction in ACh-induced vasodilatation in all groups. ACh concentration-dependant relaxation evoked a significant reduction in vasodilatory responses in HF and HFSD fetal vessels when compared to CD and SD groups (P < 0.001) (Fig. 3a). This was evident by HF weanling vessels having a larger reduction in vessels function than that of HFSD weanling vessels. CD and SD vessel responsiveness did not differ from each other. Likewise, HF and HFSD vessel vasodilatory capacity were not different from each other.

#### Weanling vessels

The combination of TRAM-34 (1 μM), Apamin (3μM), GAP-27 (100 uM) and INDO (10 μM) induced a significant reduction in Ach-induced vasodilatation in all groups. Weanlings from dams fed HF and HFSD had significantly reduced vasodilatation responses when compared to CD and SD (P < 0.001) with HF weanling vessels having a larger reduction in function than that of HFSD weanling vessels (Fig. 3b). Overall HF and HFSD were not significantly different from each other. Similarly, CD and SD vessel responsiveness was not different from each other.

#### Adult vessels

The presence of TRAM-34 (1 μM), Apamin (3 μM), GAP-27 (100 uM) and INDO (10 uM) induced a significant reduction in ACh-induced vasodilatation in all groups. A clear intermediary effect of HFSD can be observed with HF weanling vessels having a larger reduction in vessels function than that of HFSD weanling vessels. HF and HFSD mesenteric vessel responsiveness was significantly reduced when compared to all other groups (P < 0.001). HFSD offspring vessel vasodilatation was observed to be significantly reduced when compared to CD and SD offspring vessels. CD and SD mesenteric vessel function did not differ from each other (Fig. 3c).

### ACh-induced relaxation in vessels incubated with L-NAME & ODQ & INDO

#### Fetal vessels

In the presence of L-NAME (100 μM), ODQ (5 μM) and INDO (10 μM), ACh-induced relaxation was observed in a concentration-dependant manner in all fetal vessels (Fig. 4a). ACh-induced relaxation was significantly reduced in SD fetal vessels alone when compared to all other groups (P < 0.001). CD, HF and HFSD fetal vessel Ach-induced relaxation in the presence of L-NAME, ODQ and INDO were not different from one another.

#### Weanling vessels

In the presence of L-NAME (100 μM), ODQ (5 μM) and INDO (10 uM), ACh-induced relaxation was observed in a concentration-dependant manner in all fetal vessels (Fig. 4b). As observed in fetal vessels, ACh-induced relaxation was significantly reduced in SD mesenteric vessels when compared to all other groups vessel responsiveness (P < 0.001). An emerging intermediary effect of fat and salt combined can be observed in the HFSD group around −6 Log [ACh] M continuing up to −3 Log [ACh] (P < 0.001) (Fig. 4b) . Both CD and HF vessel responsiveness was not affected at this stage.

#### Adult vessels

In 135 day old male offspring mesenteric vessels in the presence of L-NAME (100 μM) and ODQ (5 μM) and INDO (10 uM), ACh-induced relaxation was observed in a concentration-dependant manner in all vessels (Fig. 4c). ACh-induced relaxation was significantly reduced in SD mesenteric vessels when compared to all other groups (P < 0.001). No significant differences were observed between CD, HF and HFSD weanling mesenteric vessels.

#### Vascular ROS production

To measure whether maternal salt and/or fat combined intake-induced changes in vascular reactivity were associated with oxidative stress levels in vascular tissue. We measured total ROS production in male adult offspring. Total ROS production was appeared to be higher in maternal fat-fed offspring vessels *per se* (supplementary figure 4). However, modest increases in ROS production in HF, SD and HFSD offspring vessels were not significantly different between groups. No effect of maternal diet and no interaction effects were observed.

#### Differential mRNA expression in vessels

**Figure 5:**
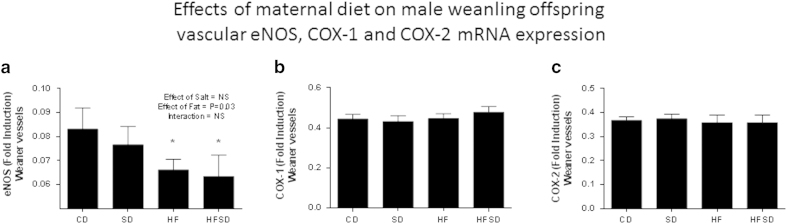
Effects of maternal diet on mRNA expression in male weanling offspring vessels. (**a**) Bar graph indicates fold change in eNOS mRNA expression present in CD, SD, HF and HFSD weanling offspring vessels. (**b**) Bar graph indicates fold change in COX-1 mRNA expression present in CD, SD, HF, HFSD weanling offspring vessels. (**c**) Bar graph indicates fold change in COX-2 mRNA expression present in CD, SD, HF, HFSD weanling offspring vessels. *denotes p < 0.001 *vs.* Control offspring. All data are means ± SEM, n = 7 per group.

**Figure 6:**
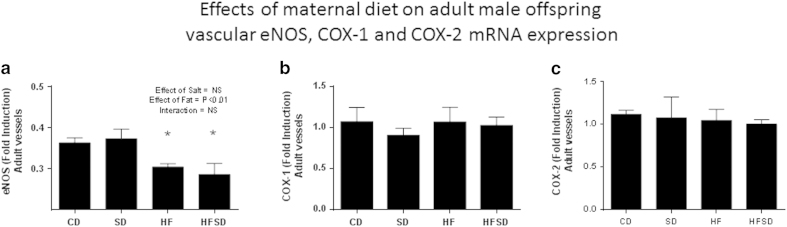
Effects of maternal diet on mRNA expression in male adult offspring vessels. (**a**) Bar graph indicates fold change in eNOS mRNA expression present in CD, SD, HF and HFSD adult offspring vessels. (**b**) Bar graph indicates fold change in COX-1 mRNA expression present in CD, SD, HF, HFSD adult offspring vessels. (**c**) Bar graph indicates fold change in COX-2 mRNA expression present in CD, SD, HF, HFSD adult offspring vessels. *denotes p < 0.001 *vs.* Control offspring. All data are means ± SEM, n = 7 per group.

An overall significant effect of fat was observed on the differential mRNA expression of eNOS in weanling and adult (P = 0.03 in weanling vessels (Fig. 5a) and P < 0.01 in adult vessels (Fig. 6a)). *Post-hoc* analysis revealed HF and HFSD male offspring having significantly lower levels of eNOS *vs.* CD and SD male offspring vessels at the fetal, weanling and adult time points. No significant differences or overall effects of maternal diet were observed in mRNA expression of COX-1 and COX-2 in both weanling and adult offspring vessels (Fig. 5b,c and Fig. 6b,c).

## Discussion

The current study describes the effects of excessive maternal fat and/or salt intake on offspring vasomotor function and cardiovascular status during developmental and into adulthood. We provide evidence that despite being fed a ‘healthy’ diet from weaning, endothelial dysfunction due to maternal dietary intake can manifest *in utero* and is persistent through-out life, culminating in a hypertensive phenotype in adult offspring. The current study, using tail cuff plethysmography confirms previous findings of increased offspring blood pressure following maternal dietary challenges, when measured by blood pressure telemetry^13^,^14^,^15^. The significant increases in resting blood pressure observed in the maternal fat, salt and slat/fat combined groups are of major importance when considering the present day global public health burden of hypertension and subsequent CVD risk, the elevation in offspring resting blood pressure is of major concern. In terms of predisposition to CVD and when considering the original findings of Barker *et al.* and the association between birth weight, elevations in adult BP (<2 mmHG) and cardiovascular mortality during adult life^6^,^7^.

We report endothelial dysfunction in offspring from either maternal salt-fed or fat-fed mothers, observing reductions in nitric oxide pathways and EDHF pathways^19^,^20^,^21^,^22^,^23^,^24^. Sofola *et al.* showed that a 8% salt diet fed to Sprague Dawley weanlings caused an increase in SBP and endothelial dysfunction, which was mediated by EDHF mechanisms rather than endothelial derived NO^25^. The current study indicates that hypertension in HF and SD fetal, weanling and adult offspring are mediated by a perturbation of differing vasodilatory mechanisms. Maternal fat intake was observed to have larger effects on nitric oxide driven pathways and a non-significant increase in vascular reactive oxygen species, in comparison to maternal salt-fed offspring which were observed to have an impaired EDHF pathway of vascular function. Interestingly, no effect on prostanoid pathways, on either vessel function or COX-1 and COX-2 gene expression was observed between diets in weaning and adult offspring vasculature. Our work and the work of others have shown reductions in endothelial function and, in the case of maternal fat-fed adult offspring, the nitric oxide pathway^19^,^20^,^21^. Endothelial dysfunction may be a consequence of activation of inflammatory pathways as a result of increased adiposity in the maturing offspring and it has been reported that, in aged rats with increased adiposity, insulin resistance may also be playing a role in increasing basal blood pressure and vascular dysfunction observed in these offspring^26^,^27^. Furthermore, there is evidence to suggest that children of obese mothers may be predisposed to hypertension in early life due to altered autonomic control and fetal cardiac sympathovagal activation^28^. Similar to results presented here, in an animal model of maternal high-fat intake, Poston *et al.* observed increased adiposity, hypertension and endothelial dysfunction in young offspring. In the rat, further results were reported by the same group, showing an increase in blood pressure as early as 30 days of age, independent of increased fat mass^29^. However, these potential mechanisms involved are difficult to elucidate.

Given that the offspring had no direct exposure to the experimental diets, the vertically transmitted vascular and hypertensive phenotypes observed in HF and SD offspring are of major concern to pregnant women and those considering pregnancy. In the case of maternal salt intake, this may be due to inhibitory action of sodium on mechanisms critical to cellular differentiation during fetal development. Previous studies investigating the effects of maternal salt intake on offspring cardiovascular status and *in vitro* studies looking at branching organogenesis in the presence of salt suggest a possible mechanism by which salt impairs cardiovascular development in fetus could be *via* an alteration in maternal plasma sodium. Therefore, a high salt intake may be impairing vascular function by raising plasma sodium, osmolality and potential for reduced branching angiogenesis *in utero* and adulthood^14^,^30^. However, the cellular mechanisms are not well described and developmental programming of hypertension and vascular function will involve a myriad of mechanistic pathways. Responses to maternal HFSD, for example, are likely to include the decreased bioavailability of NO and/or decreased capacity of EDHF transfer due to vascular remodelling during fetal development. Nevertheless, our findings indicate that maternal salt intake impairs EDHF and increased maternal fat intake impairs NO-dependant hyperpolarization and vascular eNOS gene expression with a combination of both pathways being perturbed, without the hypothesised, additive effect of salt and fat on fetal, weanling and adult offspring vessels and blood pressure *per se*.

The effects of maternal salt and/or fat intake on offspring cell number, function, tissue remodelling and future cardiovascular phenotypes are proposed to be determined during early *in utero* development. Therefore, it is possible to predict there will be a number of ‘critical windows’ during which maternal nutritional status and environment may be exerting deterimental effects on fetal vascular development. In context, nutritional programming of vascular function and adult hypertension via excessive maternal salt and/or fat intake could, in part, explain a proportion of the current global trend of increased essential hypertension and associated CVD risk, particularly with a chronically high salt, high fat Western diet observed throughout developed countries. Governments worldwide are being encouraged to reduce population salt intake to 5 g/day, to reduce overall hypertensive cases and associated cardiovascular morbidity^31^,^32^. Studies have reported the benefits of reducing salt intake or resting blood pressure by 3 mmHg can result in major reductions health care expenditure and improvements in public health^33^. Asaria *et al.* reported that, based upon a 10 year period in low to middle income countries, a 15% reduction in salt intake could prevent 8.5 million cardiovascular-related deaths^34^. Taken together, these studies provide evidence that reducing salt and/or fat intake during pregnancy may be important in lowering the global CVD burden and reducing CVD risk in future generations.

The present study suggest that moderately increased maternal salt and fat intake, and a combination of the two, may be facilitating an inhibition of vascular function and altered vascular gene expression, in terms of endothelial function and associated hypertension, which is present in young offspring and persists into adult life. Our results show consistent endothelial dysfunction and age-related decline in vascular compliance and hypertension in offspring following exposure to excessive high fat and/or salt *in utero*. The resulting decrease in NO bioavailability, EDHF transport amongst maternal dietary groups are likely to involve similar vasodilatory mechanisms which is sufficient to compromise the endothelium-dependent regulation of vascular tone leading to a persistent elevation in vascular tone and blood pressure throughout life in to adulthood.

## Author Contributions

Conceived and designed experiments: CG and MHV. Performed experiments: CG, CJH and CMR. Wrote manuscript: CG. Edited manuscript: CMR, CJH, SAS and MHV.

## Additional Information

**How to cite this article**: Gray, C. *et al*. Maternal salt and fat intake causes hypertension and sustained endothelial dysfunction in fetal, weanling and adult male resistance vessels. *Sci. Rep.* **5**, 9753; doi: 10.1038/srep09753 (2015).

## Supplementary Material

Supplementary Information
