# Supplementary material for: Maternal salt and fat intake causes hypertension and sustained endothelial dysfunction in fetal, weanling and adult male resistance vessels
Source: Sci Rep. 2015 May 8;5:9753. doi: 10.1038/srep09753 (PMC4424661; doi:10.1038/srep09753)
Supplement: Supplementary Information [file srep09753-s1.pdf]

Maternal salt and fat intake causes hypertension and sustained endothelial dysfunction in fetal, weanling and adult male resistance vessels

Clint Gray, Claudia J Harrison, Stephanie A Segovia, Clare M Reynolds, Mark H Vickers.

Supplementary Information

Supplementary figure 1

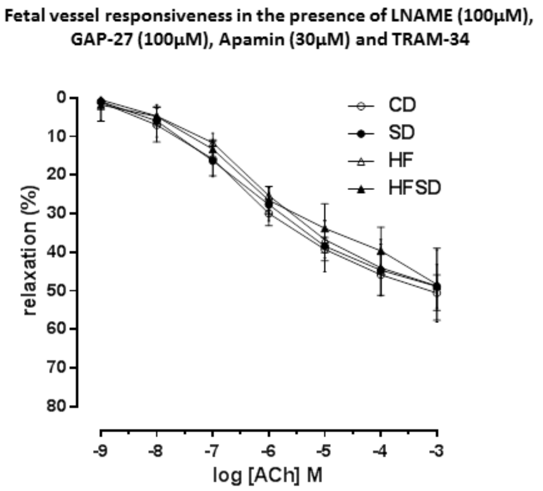

Supplementary Figure 2

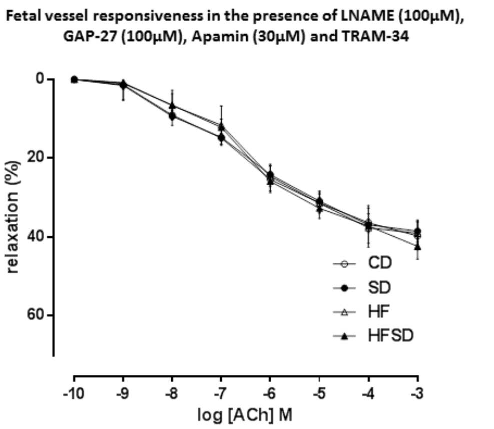

Supplementary Figure 3

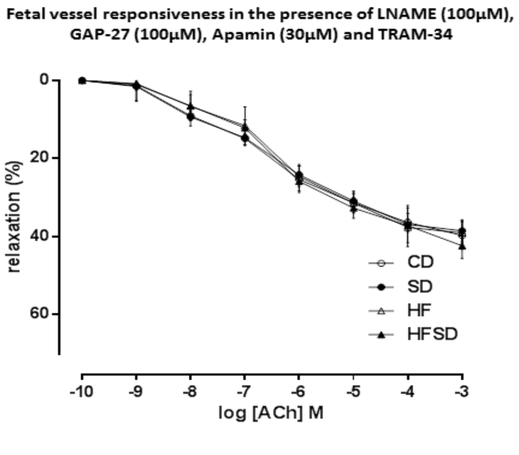

Supplementary Figure 4

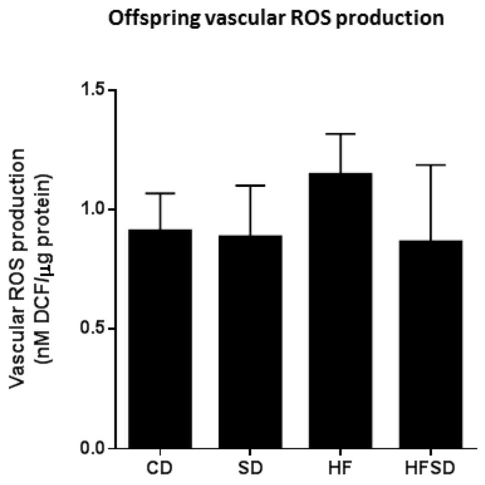

Supplementary Figure Legend

S.1

Fetal vessel responsiveness following cumulative additions of vasodilator ACh measured as % change from initial resting diameter after pre-constriction with PE (10µM) in the presence of LNAME (100µM), TRAM-34 (1µM), Apamin (3µM) and GAP-27 (100uM). All data are means ± SEM. Different dietary groups are defined as; Control group = CD, Salt group = SD, High fat group = HF and Salt and Fat combined group = HFSD, n=10/group.

S.2

Weanling vessel responsiveness following cumulative additions of vasodilator ACh measured as % change from initial resting diameter after pre-constriction with PE (10µM) in the presence of LNAME (100µM), TRAM-34 (1µM), Apamin (3µM) and GAP-27 (100uM). All data are means ± SEM. Different dietary groups are defined as; Control group = CD, Salt group = SD, High fat group = HF and Salt and Fat combined group = HFSD, n=10/group.

S.3

Adult vessel responsiveness following cumulative additions of vasodilator ACh measured as % change from initial resting diameter after pre-constriction with PE (10µM) in the presence of LNAME (100µM), TRAM-34 (1µM), Apamin (3µM) and GAP-27 (100uM). All data are means ± SEM. Different dietary groups are defined as; Control group = CD, Salt group = SD, High fat group = HF and Salt and Fat combined group = HFSD, n=10/group.

S.4

Effect of maternal diet on reactive oxygen species (ROS) production in mesenteric vessel segments. Total ROS production was measured in mesenteric segments isolated from CD, SD, HF and HFSD groups of adult male offspring. All data are expressed as mean ±SEM from 6 offspring/group. DCF indicates 2’,7’-dichlorodihydrofluorescein.
